# High-throughput screening with the *Eimeria tenella* CDC2-related kinase2/cyclin complex EtCRK2/EtCYC3a

**DOI:** 10.1099/mic.0.059428-0

**Published:** 2012-09

**Authors:** María L. Suárez Fernández, Kristin K. Engels, Frank Bender, Michael Gassel, Richard J. Marhöfer, Jeremy C. Mottram, Paul M. Selzer

**Affiliations:** 1 Intervet Innovation GmbH, Zur Propstei, 55270 Schwabenheim, Germany; 2 Institute of Microbiology and Wine Research, Johannes-Gutenberg-Universität Mainz, Becherweg 15, 55099 Mainz, Germany; 3 Wellcome Trust Centre for Molecular Parasitology, Institute of Infection, Immunity and Inflammation, College of Medical, Veterinary and Life Sciences, University of Glasgow, 120 University Place, Glasgow G12 8TA, UK; 4 Interfaculty Institute of Biochemistry, Eberhard Karls University Tübingen, Hoppe-Seyler-Str. 4, 72076 Tübingen, Germany

## Abstract

The poultry disease coccidiosis, caused by infection with *Eimeria* spp. apicomplexan parasites, is responsible for enormous economic losses to the global poultry industry. The rapid increase of resistance to therapeutic agents, as well as the expense of vaccination with live attenuated vaccines, requires the development of new effective treatments for coccidiosis. Because of their key regulatory function in the eukaryotic cell cycle, cyclin-dependent kinases (CDKs) are prominent drug targets. The *Eimeria tenella* CDC2-related kinase 2 (EtCRK2) is a validated drug target that can be activated *in vitro* by the CDK activator XlRINGO (*Xenopus laevis* rapid inducer of G2/M progression in oocytes). Bioinformatics analyses revealed four putative *E. tenella* cyclins (EtCYCs) that are closely related to cyclins found in the human apicomplexan parasite *Plasmodium falciparum*. EtCYC3a was cloned, expressed in *Escherichia coli* and purified in a complex with EtCRK2. Using the non-radioactive time-resolved fluorescence energy transfer (TR-FRET) assay, we demonstrated the ability of EtCYC3a to activate EtCRK2 as shown previously for XlRINGO. The EtCRK2/EtCYC3a complex was used for a combined *in vitro* and *in silico* high-throughput screening approach, which resulted in three lead structures, a naphthoquinone, an 8-hydroxyquinoline and a 2-pyrimidinyl-aminopiperidine-propane-2-ol. This constitutes a promising starting point for the subsequent lead optimization phase and the development of novel anticoccidial drugs.

## Introduction

The obligate intracellular apicomplexan parasites cause devastating diseases in humans and domestic animals. Well-known members of this phylum are *Plasmodium falciparum*, *Toxoplasma gondii*, *Cryptosporidium parvum*, *Theileria annulata* and *Eimeria tenella* (Mehlhorn, 2008; Morrison, 2009). The poultry intestinal disease known as coccidiosis is caused by *Eimeria* spp. such as *Eimeria acervulina*, *Eimeria necatrix*, *E. tenella* and *Eimeria mivati*. The most pathogenic species, *E. tenella*, provokes a haemorrhagic diarrhoea in young chickens, leading to a loss of weight and appetite, resorption problems, bacterial secondary infections and often also to the bird’s death (Chauhan & Roy, 2007; Mehlhorn, 2008; Shirley *et al.*, 2007). Moreover, coccidiosis causes tremendous economic losses, in excess of three billion US Dollars annually, to the world poultry industry (Dalloul & Lillehoj, 2006; Shirley *et al.*, 2007). Although prophylaxis using live-attenuated vaccines is efficient, it may have transitory adverse effects on chicken growth rate and is economically restrictive due to the high costs of these vaccines (Shirley & Bedrník, 1997; Shirley, 2000; Shirley *et al.*, 2005; Wallach *et al.*, 2008). Several drugs are available for the treatment of coccidiosis; however, resistance develops frequently (Chapman, 1997; Williams *et al.*, 1999). Therefore, there is a clear need for new, affordable and effective anticoccidial drugs (Williams *et al.*, 1999; Dalloul & Lillehoj, 2006; Shirley *et al.*, 2007).

The apicomplexan life cycle involves both asexual (sporogony and schizogony) and sexual (gamogony) reproduction (Mehlhorn, 2008). During schizogony the *Eimeria* parasite proliferates asexually with a very high cell division rate within the host cells (Kinnaird *et al.*, 2004; Mehlhorn, 2008). Evidence for the presence of cyclin-dependent kinases (CDKs) in apicomplexa (Doerig *et al.*, 2000) including *Eimeria* (Kinnaird *et al.*, 2004) suggests a similar CDK-dependent mode of cell cycle regulation to that found in higher eukaryotes (Malumbres & Barbacid, 2009). There are a high number of anti-cancer drugs that interfere with cell cycle regulation, leading to the death of the rapidly dividing cells (Tamamori *et al.*, 1998; Doerig *et al.*, 2002; Monaco & Vallano, 2003; Malumbres & Barbacid, 2009). Therefore, we, like others, predict that the disruption of the parasite’s cell cycle will lead to its death (Engels *et al.*, 2010; Waters & Geyer, 2003).

CDKs play a key role in cell cycle progression, transcription and neuronal function (Morgan, 1997), and are therefore extremely interesting for the development of novel pharmaceuticals (Sielecki *et al.*, 2000; Waters & Geyer, 2003; Bruno *et al.*, 1992; Schang *et al.*, 2006). *E. tenella* CDC2-related kinase 2 (EtCRK2) is by analogy assumed to play a similar key role in *Eimeria* (Kinnaird *et al.*, 2004; Engels *et al.*, 2010). It has been chemically validated using the synthetic flavone flavopiridole, a specific CDK inhibitor (Dai & Grant, 2003). Flavopiridole inhibits EtCRK2 in enzyme assays with an IC_50_ of 33±10 nM and a *K*_i_ of 11±3 nM, and fully inhibits *E. tenella* schizont development at concentrations of 150 and 300 nM. Concentrations below 80 nm show no inhibitory effects, and host cell toxicity is observed at concentrations above 600 nM. Therefore, *E. tenella* CDKs are considered to be chemically validated drug targets (Engels *et al.*, 2010).

As the name implies, CDKs are dependent on activation through binding of the respective cyclin (Olashaw & Pledger, 2002). To the best of our knowledge, in contrast to other apicomplexan parasites such as *Plasmodium*, no eimerian cyclins have been reported so far.

Here, we describe the bioinformatic discovery of two partial and two complete predicted cyclin-like proteins out of the publicly available genome sequence of *E. tenella*. We were able to clone one of the identified *E. tenella* cyclins (EtCYCs) (EtCYC3a) and demonstrated that its protein product was able to activate EtCRK2, in a similar manner to that shown with the non-cyclin activator *Xenopus laevis* rapid inducer of G2/M progression in oocytes (XlRINGO) (Engels *et al.*, 2010; Ferby *et al.*, 1999). In a combined *in vitro* and *in silico* high-throughput screening approach, using real (3514 compounds) and virtual (approx. 6 000 000 compounds) compound libraries, we identified numerous hit compound structures. The most promising hits were further analysed by IC_50_ and *K*_i_ determinations, resulting in three lead structures. The three lead structures have similar activities towards EtCRK2/EtCYC3a and EtCRK2/XlRINGO. From our data we conclude that most likely EtCYC3a is a natural activator of EtCRK2.

## Methods

### 

#### Bioinformatic analysis.

Bioinformatic analyses were carried out in analogy to Engels *et al.* (2010) and run on Silicon Graphics (SGI) computers (models Origin 3200, O2, Octane2, Fuel) running the SGI operating system IRIX6.5 as well as on Dell Precision workstations (models 390 and T3400) running Red Hat Enterprise Linux 5 (RHEL 5). Publicly available *E. tenella* genome data were downloaded from the Wellcome Trust Sanger Institute (http://www.sanger.ac.uk/resources/downloads/protozoa/eimeria-tenella.html).

#### Chemoinformatic analysis.

Chemoinformatic analyses were run according to Engels *et al.* (2010). At the time of the analysis the virtual compound library comprised approximately 6×10^6^ compounds. Molecular docking was done using the docking software gold as described in Engels *et al.* (2010). Molecular clustering was done using the hierarchical clustering method of the software suite Spotfire Decision Site 9.1.1 (Tibco Software) based on the MDL Keys (MDL Information Systems; nowadays Accelrys).

### Chemicals

#### 

##### Standard CDK inhibitors.

The purity of all screening compounds used was ≥90 %, if not stated otherwise. Flavopiridole was ordered as flavopiridole hydrochloride hydrate from Sigma-Aldrich; IUPAC name 2-(2-chlorophenyl)-5,7-dihydroxy-8-[(3*S*,4*R*)-3-hydroxy-1-methyl-4-piperidinyl]-4*H*-1-benzopyran-4-one; purity ≥98 %. Indirubin-5-sulfonate was ordered from Biomol International; IUPAC name (3*Z*)-2-oxo-3-(3-oxo-1*H*-indol-2-ylidene)-1*H*-indole-5-sulfonic acid; purity ≥98 %. 10-*Z*-Hymenialdisine was ordered from Axxora; IUPAC name (4*Z*)-4-(2-amino-4-oxo-1*H*-imidazol-5-ylidene)-2-bromo-1,5,6,7-tetrahydropyrrolo[2,3-c]azepin-8-one; purity ≥95 %. Purvalanol A was ordered from Axxora; IUPAC name 2-[[6-[(3-chlorophenyl)amino]-9-isopropyl-purin-2-yl]amino]-3-methyl-butan-1-ol; purity ≥95 %. Staurosporine was ordered from Sigma-Aldrich; IUPAC name (9*S*,10*R*,11*R*,13*R*)-2,3,10,11,12,13-hexahydro-10-methoxy-9-methyl-11-(methylamino)-9,13-epoxy-1*H*,9*H*-diindolo[1,2,3-gh:3′,2′,1′-lm]pyrrolo[3,4-j][1,7]benzodiazonin-1-one; purity ≥98 %. Alsterpaullone was ordered from Axxora; IUPAC name 9-nitro-7,12-dihydro-5*H*-indolo[3,2-d][1]benzazepin-6-one; purity ≥95 %.

##### Novel identified inhibitors.

BES124764 was ordered from Maybridge Ltd; IUPAC name 1-phenylsulfanyl-3-[4-[[4-(trifluoromethyl)pyrimidin-2-yl]amino]-1-piperidyl]propane-2-ol; purity ≥85 %. BES312351 was ordered from Bridge Chem Pvt. Ltd., Mumbai, India; IUPAC name 7-amino-8-hydroxy-quinoline-5-sulfonic acid hydrate; purity ≥95 %. BES130131 was ordered from InterBioScreen Ltd, Chernogolovka, Russia; IUPAC name 2,3-dichloro-5,8-dihydroxy-6-methyl-naphthalene-1,4-dione; purity ≥95 %. BES153950 was ordered from SPECS GmbH; IUPAC name (4*Z*,5*Z*,6*E*)-4,5-bis(hydroxyimino)-6-phenacylidene-hexahydropyrimidin-2-one; purity ≥90 %. BES154393 was ordered from SPECS GmbH; IUPAC name 4-chloro-1*H*-pyrazolo[4,3-c]quinolin-3-amine; purity ≥90 %.

### Biochemical analysis

#### 

##### Isolation of *E. tenella* sporozoites.

Sporulated oocysts of *E. tenella* Houghton strain (9.6×10^5^ oocysts ml^−1^ in 4 % potassium dichromate solution) were used as the source of parasite material. Sporozoites were obtained as described earlier (Hofmann & Raether, 1990). A 200 ml volume of oocysts in potassium dichromate solution was centrifuged at 6 °C (2500 g, 3 min), resuspended in 100 ml sodium hypochlorite (Honeywell Riedel-de Haën) and stirred in this solution for no more than 10 min until a deformation in the parasite cell wall was visible (monitored by microscopy) (Hofmann & Raether, 1990). Following centrifugation (2500 g, 3 min), floating oocysts were aspirated with a vacuum pump, diluted in distilled water and again centrifuged (2500 g, 3 min) (Hofmann & Raether, 1990). This step was repeated several times to remove the residual chloride. Oocysts were diluted in Hank’s Balanced Salt Solution (HBSS; Adcock-Scientific) and fractured by mixing with glass beads (1 mm diameter, Sigma-Aldrich) on a vortex mixer until a disruption of 80 % of oocysts was detected microscopically (Hofmann & Raether, 1990). The glass beads were washed several times with buffer, and after centrifugation the sporocyst pellet was resuspended in HBSS and stored at 4 °C (Hofmann & Raether, 1990).

##### RNA isolation and cDNA production.

The isolated sporocysts were centrifuged in 2 ml Eppendorf tubes at 13 000 ***g*** for 10 min at 4 °C in a tabletop centrifuge, and the pellets were collected. A 1 ml volume of TRI Reagent (Invitrogen) was added to 100 mg of sporocysts and placed on ice. The sample was immediately homogenized with a Precellys 24 homogenizer (MO-BIO Laboratories), and the disruption of sporocysts and sporozoites was verified microscopically. The total RNA from sporozoites was prepared following the manufacturer’s instructions.

In order to produce cDNA, RT-PCRs were performed with 10 µg of total RNA using the SuperScript First-Strand Synthesis system for RT-PCR (Invitrogen) according to the manufacturer’s instructions.

##### PCR amplification of EtCYC-like 3a (EtCYC3a) from cDNA.

A full-length EtCYC3a protein sequence was identified *in silico* by blast-searching the *E. tenella* proteome with several known human and protozoan cyclins. The Etcyc3a gene was amplified *in vitro* from 2 µg *E. tenella* cDNA using Platinum *Taq* DNA Polymerase High Fidelity (Invitrogen) in the presence of 2 mM MgSO_4_ and 150 pmol of each primer (SRD, Oberursel, Germany). The primers contained *Bam*HI (forward primer, 5′-TAGGATCCATGTTGGAGGCATCCCGAGAC-3′) or *Hin*dIII (reverse primer, 5′-GGCAAGCTTAGCGCAGTGATGGTTGTG-3′) restriction sites (recognition sites underlined). The reaction was incubated at 94 °C for 2 min, 40 cycles at 94 °C for 30s, 62 °C for 1 min and 68 °C for 1 min, followed by 10 min at 68 °C. Amplified fragments were separated by agarose gel electrophoresis and isolated using the QIAquick Gel Extraction kit (Qiagen) following the manufacturer’s instructions.

After fragment isolation, the PCR product was digested with *Bam*HI and *Hin*dIII (both enzymes from Roche Diagnostics) prior to insertion into the pMALc2X vector (New England Biolabs). The resulting expression plasmid pMALc2X-Etcyc3a was amplified in *Escherichia coli* TOP10 cells (Invitrogen) and the insert was verified by sequencing. For protein expression, *Escherichia coli* BL21(DE3) competent cells (Invitrogen) were transformed with the plasmid pMALc2X-Etcyc3a.

##### Expression and purification of recombinant EtCRK2 and XlRINGO.

Recombinant EtCRK2 and XlRINGO were expressed and purified according to Engels *et al.* (2010).

##### Expression and co-purification of recombinant EtCRK2-His/maltose binding protein (MBP)-EtCYC3a complex.

Both plasmids (pQE60-Etcrk2 and pMALc2X-Etcyc3a) were transformed in *Escherichia coli* BL21(DE3) (Invitrogen). The cells were used to express the recombinant EtCRK2–His and MBP–EtCYC3a fusion proteins separately overnight at 24 °C using 0.1 mM and 1 mM IPTG, respectively. Cell pellets were harvested by centrifugation at 9000 g for 40 min. Pellets from both transformations [*Escherichia coli* BL21(DE3) pLysS/pQE60-Etcrk2 and *Escherichia coli* BL21(DE3)/pMALc2X-Etcyc3a] were pooled and disrupted together using a French press. The cell lysate was incubated in a shaker for 1 h at 4 °C to allow for the formation of an EtCRK2–His/MBP-EtCYC3a complex. The resulting complex was co-purified by amylose affinity chromatography using the MBP-tag of EtCYC3a. Free MBP was subsequently removed using size exclusion chromatography as described for MBP–XlRINGO by Engels *et al.* (2010).

##### IC_50_, *K*_i_ determination, and compound solubility.

IC_50_ and *K*_i_ determination based on the time-resolved fluorescence energy transfer (TR-FRET) assay as well as compound solubility testing was done according to Engels *et al.* (2010).

##### Liquid chromatography/mass spectrometry (LC/MS) and NMR analyses for compound quality determination.

LC/MS and NMR analyses for compound quality control were conducted as described in Engels *et al.* (2010).

## Results and Discussion

### *In silico* identification of EtCYCs

**Fig. 1.**
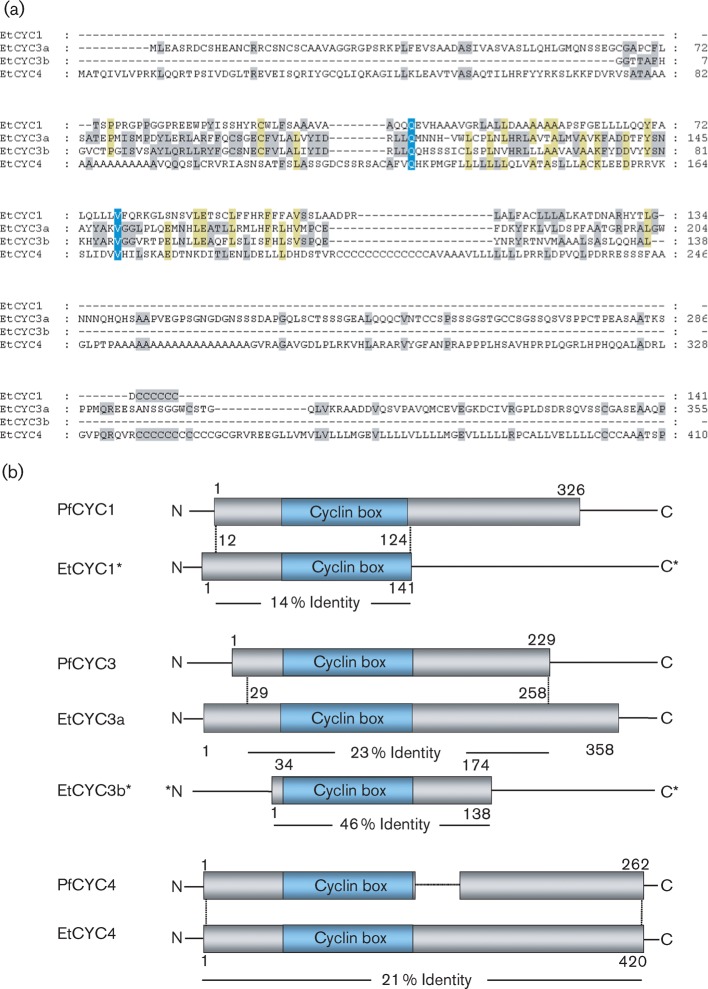
Bioinformatic identification of cyclin-like proteins of *E. tenella*. (a) Multiple sequence alignment of the potential cyclin-like *E. tenella* proteins (EtCYC1, EtCYC3a, EtCYC3b and EtCYC4). For EtCYC1 and EtCYC3b, only incomplete sequences could be identified. Blue-shaded residues are conserved among the four sequences, yellow-shaded residues among any three of the four sequences, grey-shaded residues among any two only. (b) Comparison of known cyclin-like sequences of *P. falciparum* and *E. tenella*. The potential cyclin-like proteins are homologous to the known cyclin-like proteins of *P. falciparum*. The characteristic cyclin box sequence motif is conserved among the apicomplexan cyclin-like protein sequences and is coloured blue.

In order to identify potential EtCYCs we ran a number of blast database searches on the currently unfinished genomic dataset of *E. tenella* using a wide set of cyclins, including *Plasmodium falciparum* cyclin (PfCYC)1 (Le Roch *et al.*, 2000), PfCYC2, PfCYC3 and PfCYC4 (Merckx *et al.*, 2003) from *P. falciparum*, as query sequences in analogy to Engels *et al.* (2010). The database queries revealed four potential *E. tenella* gene sequences which might encode cyclins (Fig. 1a).

(1) Query sequence PfCYC1 identified a potentially homologous gene product fragment of 141 aa, which we named EtCYC1. It was not possible to identify the full-length sequence of this hypothetical protein fragment.

(2) Query sequence PfCYC2 led to no results, i.e. no *E. tenella* gene homologous to the corresponding Pfcyc2 gene could be found.

(3) Query sequence PfCYC3 led to two significant hits, which we named EtCYC3a and EtCYC3b. While the full-length (358 aa) EtCYC3a sequence was available, only a partial sequence for EtCYC3b (138 aa) could be retrieved. The two sequences EtCYC3a and EtCYC3b share a distinct sequence identity of 66 % at the protein level over the whole available length of the EtCYC3b sequence. However, because the available EtCYC3b sequence was only about one-third the length of the corresponding EtCYC3a sequence and because the N-terminal end as well as the C-terminal end of the sequence were missing, we pursued our investigation using the full-length sequence only. The molecular size of the EtCYC3a protein was predicted to be 38 kDa.

(4) Query sequence PfCYC4 identified a full-length sequence (420 aa) which we named EtCYC4. The molecular size of this potential protein was predicted to be 44 kDa.

The four hypothetical proteins and protein fragments EtCYC1, EtCYC3a, EtCYC3b and EtCYC4 were subjected to an InterproScan analysis. A cyclin-box motif which forms the first of five α-helical repeats of the canonical cyclin fold (Jeffrey *et al.*, 1995) was identified for each of them (Fig. 1b). Other typical cyclin motifs such as the MRAIL motif, the RXL motif and the destruction box (Russo *et al.*, 1996; Schulman *et al.*, 1998; Morgan, 1997) were not found in any of the hypothetical proteins.

PfCYC4 is unable to activate PfPK5 *in vitro* and has been proposed to have roles distinct from cell cycle regulation (Nairn & Greengard, 2001; Merckx *et al.*, 2003). Although PfCYC4 and EtCYC4 are homologous to each other, an orthologous relation of the two proteins has not been proven so far. Nevertheless, in the absence of other guides to a decision, we discounted EtCYC4 from our analysis and concentrated on EtCYC1 and EtCYC3a as being potential activating partners of EtCRK2. As we were unable to access full-length EtCYC1, we focused attention on EtCYC3a as the potential activating partner of EtCRK2.

### *In vitro* activation of EtCRK2

#### Activation of EtCRK2 by XlRINGO.

**Fig. 2.**
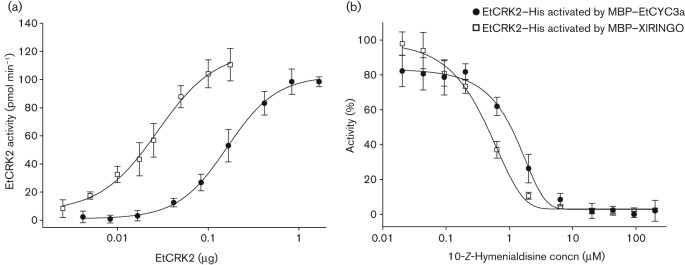
Comparison of the activity and inhibition of EtCRK2–His activated either by MBP–EtCYC3a or by MBP–XlRINGO. (a) Both activator proteins were able to activate EtCRK2 in a similar manner. Values are depicted as mean±sd EtCRK2 activity values from at least four independent experiments (*n*≥4). (b) Percentage activity of the two protein–activator complexes in the presence of increasing concentrations of 10-*Z*-hymenialdisine. Eleven inhibitor concentrations from 0.02 to 200 µM were tested in the TR-FRET assay. Points represent mean values±sd from at least three independent experiments (*n*≥3). The IC_50_ values of the inhibitor for EtCRK2/XlRINGO and EtCRK2/EtCYC3a were calculated to be 0.44±0.08 and 0.93±0.40 µM, respectively.

XlRINGO is known to be able to fully activate mammalian CDKs (Karaiskou *et al.*, 2001) as well as PfPK5 (Merckx *et al.*, 2003; Nebreda 2006). Therefore, we tested XlRINGO as an activator of EtCRK2. Using a TR-FRET assay with a fluorescein-labelled histone H1 substrate we determined the *K*_m_ value for ATP to be 9.39±1.21 µM (data not shown) and its specific activity to be 1.29±0.24 µmol min^−1^ mg^−1^ (Fig. 2a). From these data we conclude that XlRINGO is able to activate EtCRK2.

#### Activation of EtCRK2 by EtCYC3a.

**Fig. 3.**
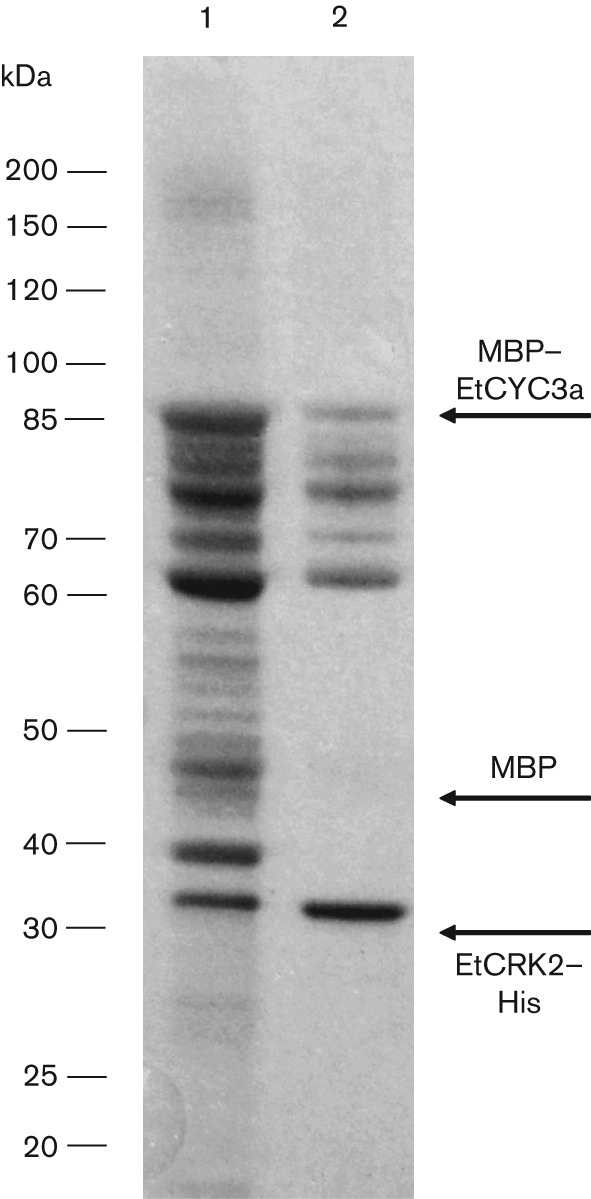
SDS-PAGE of the co-purification of the EtCRK2–His/MPB–EtCYC3a complex. Lane 1, amylase affinity chromatography, protein complex eluate fraction; lane 2, size exclusion chromatography, eluate fraction above 100 kDa.

To further study the biochemical properties of EtCYC3a and its ability to activate EtCRK2, we cloned the entire coding region into the pMALc2X vector, allowing for the expression of an EtCYC3a–MBP fusion protein (MBP–EtCYC3a) in *Escherichia coli*. Cells expressing MBP–EtCYC3a and cells expressing EtCRK2–His were pooled and disrupted together, and the cell lysate was incubated to allow for the formation of an EtCRK2–His/MBP–EtCYC3a complex. The resulting complex was co-purified by amylase affinity chromatography using the MBP-tag of EtCYC3a. If EtCRK2–His and MBP–EtCYC3a did indeed form a stable complex, this affinity chromatography step should have retained the protein complex as well as monomeric MBP–EtCYC3a, while all other components not having an MBP-tag should have been removed in the wash. A gel electrophoresis under denaturing conditions (SDS-PAGE) of the eluate fraction showed multiple proteins, including two proteins at 32 and 80 kDa, corresponding to the sizes expected for EtCRK2–His and MBP–EtCYC3a, respectively (Fig. 3, lane 1, arrows). Size exclusion chromatography was then performed and the 112 kDa complex purified (Fig. 3, lane 2). Proteins between 60 and 80 kDa correspond most likely to degradation products of MBP–EtCYC3a.

The purified EtCRK2–His/MBP–EtCYC3a (EtCRK2/EtCYC3a) complex was active in the TR-FRET assay. EtCYC3a, like XlRINGO, was able to fully activate EtCRK2 (Fig. 2a). Moreover, both complexes showed comparable IC_50_ and *K*_i_ values for standard inhibitors (Fig. 2b, Table 1). The *K*_m_ value for ATP of EtCRK2 in the EtCRK2/EtCYC3a complex was 9.05±0.76 µM (*n* = 3) and is very similar to the *K*_m_ value for ATP of EtCRK2 in the EtCRK2/XlRINGO complex (Table 2). However, its specific activity in the EtCRK2/EtCYC3a complex of 0.15±0.02 µmol min^−1^ mg^−1^ is about nine times lower than its specific activity of 1.29±0.24 µmol min^−1^ mg^−1^ in the EtCRK2/XlRINGO complex (Table 2). This is in good agreement with the findings of Merckx *et al.* (2003) that XlRINGO is a stronger activator for PfPK5 than *P. falciparum* cyclins. We therefore hypothesize that EtCYC3a is a natural activator of EtCRK2. Whether EtCYC1, EtCYC3b and EtCYC4 are also able to activate EtCRK2 has to be proven in future experiments.

**Table 1.** Comparison of IC_50_ and *K*_i_ values of standard CDK inhibitors Results are expressed as mean values±sd of at least three independent experiments (*n* = 3–6). See also Fig. 2(b).

CDK inhibitor	IC_50_ (nM)			*K*_i_ (nM)		
	EtCRK2/XlRINGO	EtCRK2/EtCYC3a	HsCDK2/HsCYCA	EtCRK2/XlRINGO	EtCRK2/EtCYC3a	HsCDK2/HsCYCA
Staurosporine	90±10	190±90	3±1	20±10	530±200	1±0
Indirubin-5′-sulfonic acid	670±180	940±310	230±110	170±30	240±100	80±10
Purvalanol A	800±110	1030±180	140±40	80±10	360±130	40±1
Alsterpaullone	220±60	640±340	160±80	70±2	210±40	60±0
Flavopiridole	40±10	260±70	50±10	10±3	260±20	20±4
10-*Z*-Hymendialdisine	440±80	930±40	120±20	50±20	190±10	30±1

**Table 2.** Comparison of *K*_m_ values and specific activity of EtCRK2 in the two protein–activator complexes Results are expressed as mean values±sd of at least three independent experiments (*n* = 3).

Parameter	EtCRK2/XlRINGO	EtCRK2/EtCYC3a
Specific activity (µmol min^−1^ mg^−1^)	1.29±0.24	0.15±0.02
*K*_m_ for ATP (µM)	9.39±1.21	9.05±0.76
*K*_m_ for fluorescein-labelled substrate (µM)	1.72±1.10	2.05±1.18

### Inhibition assays using EtCRK2 /XlRINGO and EtCRK2/EtCYC3a complexes

**Fig. 4.**
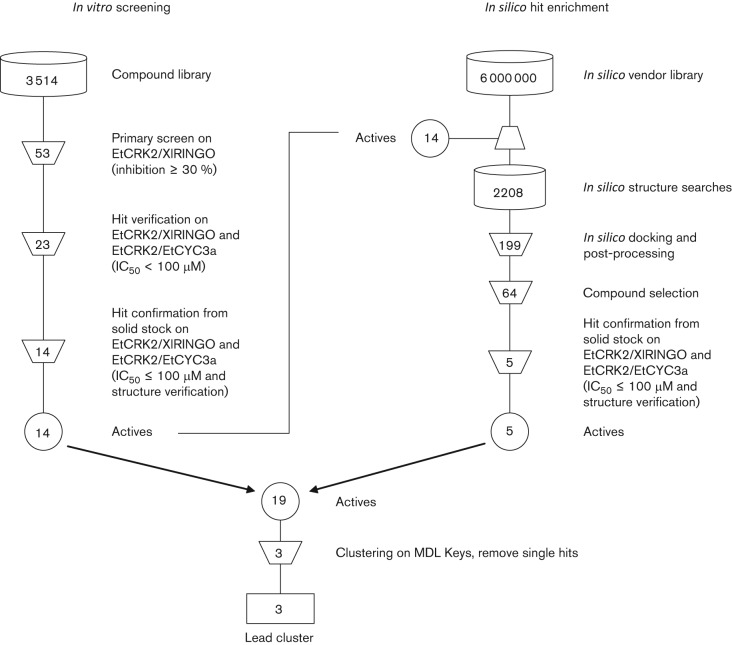
Filtering steps during *in vitro* screening and *in silico* hit enrichment. Three lead compounds, each being the most active representative of their respective cluster of structurally related compounds, were selected.

In order to discover specific inhibitors of EtCRK2 we performed an *in vitro* screening campaign supported by an *in silico* hit enrichment (Fig. 4). In a primary screening we assayed approximately 3500 compounds from a highly diverse excerpt of a vendor compound library on the active EtCRK2/XlRINGO complex. The hit threshold for this primary screen was set to 30 % inhibition at a concentration of 30 µM, achieving a hit rate of 1.5 % (53 primary hits). The primary hits were then confirmed in a secondary screen using the EtCRK2/XlRINGO complex as well as the EtCRK2/EtCYC3a complex, resulting in 23 compounds showing an IC_50_ value equal to or below 100 µM (IC_50_ ≤100 µM) on both EtCRK2 complexes. For the final confirmation step, the 23 compounds were freshly dissolved from solid stock and tested on both EtCRK2 complexes (IC_50_ ≤100 µM), and the structure of the compounds was verified using LC/MS and NMR. The activity and the structural integrity of 14 compounds were verified.

In order to allow for the discovery of additional compounds belonging to the same chemical classes we performed an *in silico* hit enrichment. Each of the 14 actives was used as a starting structure for a fingerprint-based similarity search using a Tanimoto similarity metric and a scaffold-based substructure search. The resulting virtual compound library of 2208 compounds was then screened using molecular docking and post processing as described by Engels *et al.* (2010), leading to 64 potential inhibitors of EtCRK2. These 64 compounds were purchased and tested in our *in vitro* set-up. Five compounds were confirmed to have an IC_50_ value below 100 µM (IC_50_ <100 µM) on both EtCRK2 complexes. Together with the 14 hits coming from the initial *in vitro* screen we found an overall hit set of 19 compounds. In order to identify compound families whose members belonged either to the same chemical class or to a common scaffold with similar pharmacophore features we performed a clustering based on the MDL Keys fingerprint. The clustering identified three compound clusters: the naphthoquinone cluster, the 8-hydroxyquinoline cluster, and the 2-pyrimidinyl-aminopiperidine-propane-2-ol cluster, which we named after the most active representative of the individual cluster. Naphthoquinones, 8-hydroxyquinolines as well as 2-pyrimidinyl-aminopiperidine-propane-2-ols (Table 3) have already been described in the literature as inhibitors of different kinases (Aziz *et al.*, 2008, Al-Sha’er & Taha, 2010; Kim *et al.*, 2011).

**Table 3.**
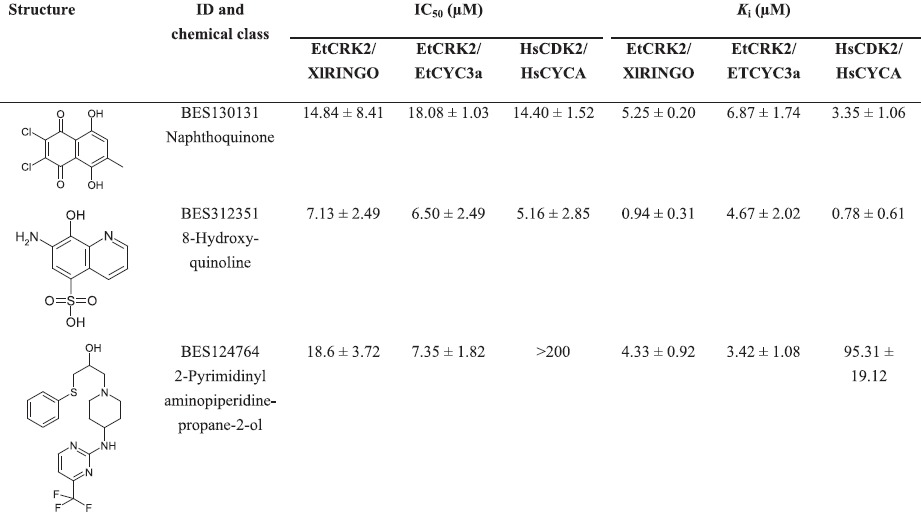
Comparison of IC_50_ and *K*_i_ values of the lead structures (most active representatives of the three clusters) Results are expressed as mean values±sd of at least three independent experiments (*n* = 3–6).

**Fig. 5.**
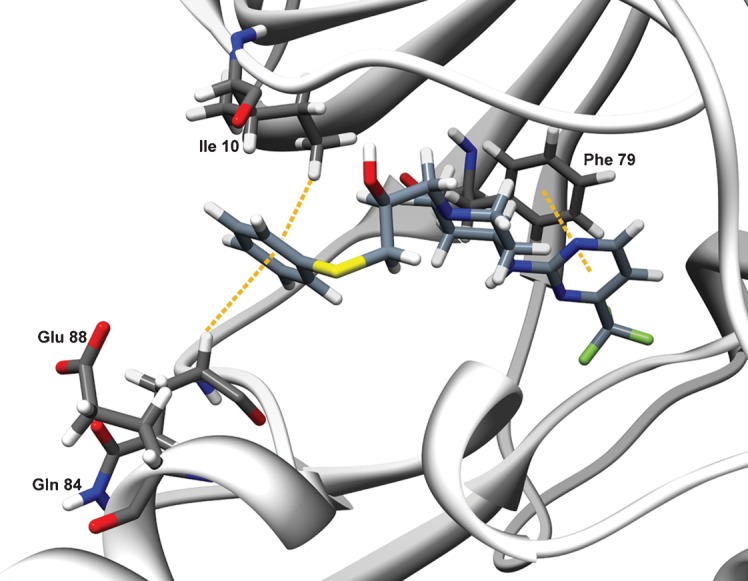
Top ranked docking solution for BES124764. BES124764 is docked into the predicted ATP binding pocket of EtCRK2. Arene–H interactions to Gln84 and Ile10 and the arene–arene interaction to Phe79 are shown as dashed orange lines.

The most active representative of each of the three clusters was subjected to *K*_i_ value and IC_50_ determinations on both EtCRK2/XlRINGO and EtCRK2/EtCYC3a, as well as on the complex of human CDK2 activated by human cyclin A: HsCDK2/HsCYCA (Table 3). While the naphthoquinone representative and the 8-hydroxyquinoline representative displayed similar activities towards the EtCRK2 complexes and HsCDK2/HsCYCA, BES124764, the 2-pyrimidinyl-aminopiperidine-propane-2-ol representative, was approximately 25–30 times more active towards the two *E. tenella* complexes than towards the HsCDK2/HsCYCA complex. The *K*_i_ determination suggests ATP competitiveness, which in addition is backed by the molecular binding mode found in docking experiments (Fig. 5). From its molecular binding mode, BES124764 is an atypical kinase inhibitor forming no H-bonds to the hinge region residues (Fig. 5). Moreover, the selectivity site E88K reported by Engels *et al.* (2010) is not addressed by this inhibitor, and it is therefore not clear how the selectivity for the eimerian enzyme is conferred. Therefore, we will focus future work towards the molecular mode of action of BES124764 and its derivatives.

### Conclusion

EtCRK2 is a valid drug target for the apicomplexan parasite *E. tenella* (Engels *et al.*, 2010). Although those authors were able to assay EtCRK2 using the non-native activator XlRINGO, the native activator of EtCRK2 was not known. Therefore, one part of our work was devoted to the identification of the putative native activator of EtCRK2. Using bioinformatics and molecular biology methods we successfully identified a number of potential cyclin-like proteins which could be the native activators. The most promising of these proteins, EtCYC3a, was studied further and it was proven that it is able to activate EtCRK2.

In a subsequent *in vitro* screening on EtCRK2/XlRINGO and EtCRK2/EtCYC3a supported by an *in silico* hit enrichment, we were able to identify 19 active compounds. We subsequently identified three clusters of structurally related compounds, which we named after the most active representative of the cluster as the naphthoquinone cluster, 8-hydroxyquinoline cluster and 2-pyrimidinyl-aminopiperidine-propane-2-ol cluster. The most active representative of each of the three clusters was picked as a lead structure and was assayed for its selectivity for EtCRK2 versus HsCDK2. It turned out that BES124764, the representative of the 2-pyrimidinyl-aminopiperidine-propane-2-ol cluster, was selective for EtCRK2. Therefore, BES124764 and derivatives thereof will be introduced into lead optimization, the next step of the stage-gated drug discovery process as described by Rohwer *et al.* (2011). In the next steps of this process, the compounds will be chemically modified and carefully evaluated for their specificity against several CDK and CDK-like proteins of mammalians, and for their activity towards parasites in cell culture and *in vivo*.
